# National Birth Defects Prevention Month and Folic Acid Awareness Week — January 2013

**Published:** 2013-01-11

January is National Birth Defects Prevention Month. Each year, birth defects affect approximately one in 33 newborns in the United States (1). Birth defects are a leading cause of infant mortality, accounting for approximately 20% of infant deaths (2). Babies who survive and live with birth defects are more likely to have life-long physical and cognitive challenges. In the United States each year, the total hospital costs of children with birth defects exceed $2.6 billion (3).

Evidence suggests that use of tobacco or alcohol (4,5), uncontrolled diabetes (6), failure to consume 400 *μ*g of folic acid daily (7), and failure to achieve and maintain a healthy weight before and during pregnancy (8) might be associated with birth defects. Health-care professionals can help prevent birth defects by encouraging women of childbearing age to manage health conditions and adopt healthy behaviors before becoming pregnant. Additional information is available at http://www.cdc.gov/birthdefects.

January 6–12, 2013, is National Folic Acid Awareness Week. CDC urges all women of childbearing age who are capable of becoming pregnant to consume 400 *μ*g of folic acid every day, before becoming pregnant and during pregnancy, to help reduce the risk for neural tube defects (major birth defects of the brain and spine) (7). Health-care providers should encourage women to consume folic acid in fortified foods or supplements, or a combination of the two, in addition to a varied diet rich in folate. Additional information about folic acid is available at http://www.cdc.gov/folicacid.
